# Antinociceptive effect of methanol extract of *Celosia cristata* Linn. in mice

**DOI:** 10.1186/s12906-016-1393-5

**Published:** 2016-10-22

**Authors:** Shanta Islam, Md Shafiullah Shajib, Tajnin Ahmed

**Affiliations:** Department of Pharmacy, Stamford University Bangladesh, 51 Siddeswari Road, 1217 Dhaka, Bangladesh

**Keywords:** *Celosia cristata* Linn, Amaranthaceae, Medicinal plant, Antinociceptive, Opioid system

## Abstract

**Background:**

*Celosia cristata* Linn. (Amaranthaceae) is used in traditional medicine for the treatment of headache, sores, ulcers, eye inflammations, skin eruption, painful menstruation and carpal tunnel syndrome. This study was performed to evaluate the antinociceptive activity of methanol extract of the whole plant of *C. cristata* (MECC).

**Methods:**

The evaluation of the antinociceptive effect of MECC was performed using thermal (hot plate, tail immersion test) and chemical (acetic acid, formalin, and glutamate-induced nociception test) pain models in mice at four different doses (50, 100, 200, 400 mg/kg; p.o.). Involvement of opioid receptors mediated central antinociceptive mechanism of MECC was evaluated using naloxone. Furthermore, the association of ATP-sensitive K^+^ channel and cGMP pathway were evaluated using glibenclamide and methylene blue respectively.

**Results:**

Oral treatment of MECC produced significant, strong and dose-dependent central and peripheral antinociceptive effect in experimental pain models. MECC significantly increased the latency time of thermal threshold in both hot plate and tail immersion test. The inhibition of writhing syndrome by the extract in the acetic acid-induced writhing test was remarkable. MECC significantly reduced the formalin-induced neurogenic and inflammatory pain. In addition, the inhibition of glutamate-induced paw licking and edema by MECC was significant. The antinociceptive effect was significantly reversed by naloxone and glibenclamide, suggesting the association of opioid and ATP-sensitive K^+^ channel system respectively. In addition, MECC also demonstrated the involvement of cGMP pathway in the antinociceptive action.

**Conclusion:**

The study suggests that *C. cristata* possess significant antinociceptive effect which is associated with both central and peripheral mechanisms and provides a rationale for its extensive use at different painful conditions in traditional medicine.

## Background

*Celosia cristata* Linn. (commonly known as Cockscomb) is an annual herb from Amaranthaceae family. It is locally called ‘Moragphul’ and grown in the garden as an ornamental plant in Bangladesh. The plant is applied for the treatment of headache, sores, ulcers, eye inflammations, skin eruption, painful menstruation and carpal tunnel syndrome in ethnomedicine [1–4]. Flower of the plant is used in the treatment of abdominal pain, epistaxis, hemoptysis, hematuria, hematemesis and painful bones [5–7]. Leaves are used in cuts, wounds and body swelling [8, 9]. Seeds are applied in the treatment of mouth sores, inflammation of the ciliary body, cornea and iris and piles [6, 10]. Branches and roots are used in leucorrhea [11].

The plant contains asparagine, asparagine-linked glycon, protein, glycoproteins [2]. Hyaluronic acid (HA) has been found in the plant [12]. The aerial parts of the plant contain cristatein and tlatlancuayin [13]. Seeds of the plant have been reported to contain 5-hydroxy-7-methoxyflavone, 5-methoxy-6,7-methylenedioxyflavone, 5-hydroxy-6,7-dimethoxyflavone 5,7-dimethoxyflavone, cochliophilin A, kaempferol, stigmasterol, β –sitosterol, 4-hydroxyphenethyl alcohol, 2-hydroxyoctadecanoic acid, saponins named celosin A, B, C and D, cristatain and semenoside A [13–16]. Antiviral glycoproteins named CCP-25 and CCP-27 have been found in leaves [17]. Betanin has been found in the callus line of the plant [18]. In vivo and in-vitro studies reported anti-inflammatory activity of betanin [19]. The isolated compound, HA, has been reported to effective against inflammations like dermal, corneal wound and osteoarthritis [20, 21]. Tlatlancuayin has been reported to possess cell renewal and antioxidant properties [22]. Cristatain and semenoside A, isolated from seeds, have been reported to exhibit hepatoprotective activity in mice [15, 16]. Pharmacological investigations reported the antioxidant, anti-aging, adipogenesis reduction and acetylcholinesterase, butyrylcholinesterase, tyrosinase enzyme inhibition activity of the plant [23–25]. In vivo studies of the flower of the plant in mice and rabbits showed significant hemostatic action. Leaves of the plant have been reported to possess anthelmintic activity [26]. In vivo study of the leaves demonstrated the suppression of development of benign prostate hyperplasia (BPH), a disease associated with oxidative stress and inflammatory process [27]. In vitro study of the crude extract of the plant exhibited anti-inflammatory activity by suppression of histamine release and arachidonic acid synthesis [28].

The application of *C. cristata* in different painful states in traditional medicine, anti-inflammatory action of its crude extract as well as isolated compounds and lack of scientific report regarding its antinociceptive action prompted us to conduct the present study to evaluate the effect of methanol extract of *C. cristata* (MECC) whole plant using different thermal and chemical nociceptive models in mice.

## Methods

### Plant material and extraction

*Celosia cristata* were collected from the Gowsul Azam Nursery, Kamalapur, Dhaka, Bangladesh on November 27, 2014. The collected plants were identified by Bushra Khan, Principal Scientific Officer, Bangladesh National Herbarium, Mirpur, Dhaka, Bangladesh and a voucher specimen (DACB: 41890) has been deposited for further reference. The plant material was shade dried and grounded. Then 205 g of powdered material were macerated by 1000 mL of methanol. The solution was occasionally stirred at 25 ± 2 °C for 7 days and then filtered using sterilized cotton and Buchner funnel. The filtrate was concentrated to evaporate solvent using rotary evaporator at 40 °C and 50 r.p.m. Finally, 14.26 g (yield 6.96 %) of dried extract was obtained and this crude extract was used for phytochemical screening, toxicity, and antinociceptive activity studies.

### Chemicals and drugs

The chemicals and drugs used in the present study are methanol, toluene, ethyl acetate, dichloromethane, vanillin, sulphuric acid, Folin–Ciocalteu’s reagent, aluminum chloride, Na-K tartrate, formalin, acetic acid, L-glutamic acid, (Merck Co., Darmstadt, Germany), DPPH (2,2-Diphenyl-1-picrylhydrazyl), ascorbic acid, quercetin, stigmasterol, methylene blue, pentobarbital sodium (Sigma Co., St. Louis, MO, USA), digoxin (Aristopharma Ltd., Shampur, Dhaka, Bangladesh), atropine, morphine sulfate (Gonoshasthaya Pharmaceuticals Ltd., Savar, Dhaka, Bangladesh), diclofenac sodium (Novartis Bangladesh Ltd., Gazipur, Dhaka, Bangladesh), glibenclamide (Square Pharmaceuticals Ltd., Gazipur, Dhaka, Bangladesh), naloxone hydrochloride (Samarth Life Sciences Pvt. Ltd., Nalagarh, Himachal Pradesh, India).

### Animals

Swiss albino male mice with 20–25 g body weight (b.w.) were collected from Animal Resources Branch of the International Center for Diarrhoeal Disease Research, Bangladesh (icddr,b) The animals were acclimatized for 14 days in laboratory condition before experiments. The animals were housed in 120 × 30 × 30 cm cages at standard laboratory environment (room temperature 25 ± 2 °C; relative humidity 55–60 %; 12 h light-dark cycle) and were provided with standard diet (icddr,b formulated) and tap water *ad libitum*. Flake wood shavings were used for bedding. Health status of animals was monitored every day. The animals were randomly selected and divided into control, positive control and experimental group (*n* = 5) for each experiment. The animals were abstained from food overnight only before experiments. All the experiments were carried out in accordance with The Swiss Academy of Medical Sciences and the Swiss Academy of Sciences formulated *Ethical Principles and Guidelines for Scientific Experiments on Animals* (1995) and performed under the approval of Ethics Committee of Stamford University Bangladesh (SUB/IAEC/14.07). The acute oral toxicity test was carried out following the guideline (420 – fixed dose procedure) of Organization for Economic Cooperation and Development (OECD). The animals were euthanized using pentobarbital in accordance with AVMA guidelines for the Euthanasia of Animals: 2013 Edition and made all efforts to alleviate animal suffering.

### Drugs and treatments

The control group orally received vehicle (0.9 % sodium chloride) at the dose of 10 mL/kg (b.w.) 30 min before the experiments. The positive control group intraperitoneally (i.p.) received standard drug morphine in hot plate, tail immersion and formalin-induced licking test at the dose of 5 mg/kg and diclofenac sodium in acetic acid-induced writhing and glutamate-induced paw licking test at the dose 10 mg/kg 15 min before the experiments. MECC was administered orally at the doses of 50, 100, 200, 400 mg/kg (b.w.) 30 min before the experiments. The doses of MECC were selected from trial and previously reported effective doses of *C. cristata* [29]. To evaluate the involvement of opioid-mediated antinociceptive activity, naloxone was administered (i.p.) at the dose of 2 mg/kg 15 min before morphine sulfate or MECC administration in the hot plate and tail immersion test. Methylene blue (20 mg/kg) and glibenclamide (10 mg/kg) were intraperitoneally employed 15 min before vehicle or MECC (50, 100, 200 and 400 mg/kg) administration to evaluate the involvement of cyclic guanosine monophosphate (cGMP) and ATP-sensitive K^+^ channel pathway respectively. All the doses of drugs and MECC were prepared using the vehicle.

### Phytochemical analysis

#### Phytochemical screening

MECC was qualitatively tested for the detection of proteins, carbohydrates, steroids, saponins, alkaloids, flavonoids, tannins, glycosides and resins following standard procedures [30]. The phytoconstituents of MECC was further authenticated by thin layer chromatography (TLC). Aliquots of (10 % w/v dissolved in methanol) MECC extract was spotted on pre-coated silica gel 60 F_254_ plates (Merck Co., Darmstadt, Germany). The phytochemical profile was obtained using different solvent systems as flavonoids, phenolics and steroids (toluene: ethyl acetate: acetic acid (9:1:1); glycosides (ethyl acetate: methanol (10:1); alkaloid (dichloromethane: methanol (8:1). A collection of standard phytochemicals such as quercetin, gallic acid, stigmasterol, digoxin, and atropine were co-chromatographed for flavonoids, phenolics, steroids, glycosides, alkaloids respectively. The developed chromatographed plates were visualized under visible (254 nm), UV light (356 nm) or using suitable spraying reagents (such as steroid by 1 % w/v vanillin-sulphuric acid solution, glycosides by sulphuric acid, alkaloid by Dragendorff’s reagent) [31]. Then retention factor (R_f_) for each spot was determined as R_f_ = distance travelled by the solute/distance travelled by the solvent). Each experiment was carried out twice and the mean R_f_ values were compared with the standard markers.

#### Determination of phenolic content

The total phenolic content (TPC) was determined using Folin–Ciocalteu’s reagent [32]. 1 mL of MECC (200 μg/mL) was mixed with 0.5 mL of Folin–Ciocalteu’s reagent. 5 min later 4 mL of sodium carbonate (7.5 % w/v in distilled water) solution was added to the mixture. The solution was incubated at 20 °C for 60 min. The absorbance of the solution was read at 765 nm using spectrophotometer (Specord 250, Analytik Jena, Germany). A calibration curve (y = 0.0057x + 0.0146, *R* ^2^ = 0.9985) was constructed by preparing gallic acid solutions (25 – 400 mg/L). The experiment was carried out in triplicate and the mean value of absorbance was calculated. Then TPC was determined in gallic acid equivalents (GAE) from the following formula: A = (C × V)/m, where, A denote total phenolic content of extract equivalent to gallic acid, C is the concentration of the gallic acid obtained from calibration curve (mg/mL), V denote volume of the extract (mL) and m is the plant extract weight (g).

#### Determination of flavonoid content

Total flavonoid content was determined using aluminum chloride (AlCl_3_) [33]. 1 ml of MECC (100 μg/mL) was taken and mixed with 2 mL of methanol. Then 0.1 mL of 10 % AlCl_3_ (w/v in distilled water), Na-K tartrate (1 M) solution and 2.8 mL distilled water was sequentially added to the extract solution. Then the mixture was vigorously shaken by vortex mixture (Clifton CM-1, Camlab, UK) and incubated at room temperature for 30 min. The absorbance of the solution was taken using spectrophotometer at 415 nm. The experiment was carried out in triplicate and the mean absorbance value was noted. The total flavonoid content was measured from the calibration curve of quercetin (y = 0.0165x + 0.1353, *R* ^2^ = 0.9933) and expressed as mg of quercetin equivalent/g of plant extract.

#### DPPH free radical scavenging assay

To determine the scavenging effect of MECC on DPPH radical, a stock solution of 1.6 mg extract in 0.4 mL methanol was prepared. The test solution was prepared at the concentration of 1.5625, 3.125, 6.25, 12.5, 25, 50, 100, 200 and 400 μg/mL using methanol. A volume of 2 mL test solution was added to 2 mL of a methanol solution of DPPH (0.1 mM). The mixture was properly mixed and kept in a dark place at room temperature for 30 min. The absorbance of the solutions was read at 517 nm against blank. The experiment was carried out triplicate for each concentration. The percentage of inhibition of DPPH free radicals was calculated from following equation: % inhibition = [(absorbance of blank – absorbance of the sample)/absorbance of blank] × 100. The scavenging capacity of ascorbic acid in the same concentration was determined similarly as standard [34]. The concentration at which the 50 % DPPH free radicals were inhibited (IC_50_) was calculated using GraphPad prism, Version 6.05.

### Acute toxicity test

The animals were divided into control and three experimental groups (*n* = 5). MECC was orally administered at the dose of 1000, 2000 and 4000 mg/kg to the experimental groups. The animals were kept in a distinct cage. After gavage, animals were provided free access to water *ad libitum* and food. Any allergic reactions (skin rashes, itching), discharges from eyes and mucous membrane, behavioral changes, food and water refusal, salivation, convulsion, tremors, diarrhea, and mortality of the animals were observed for 14 days. After the observation period, the body weight of surviving animals was recorded. Then, animals were sacrificed to examine any abnormalities and significant gross changes of the vital organs [35, 36].

### Pharmacological tests

#### Hot plate test

Hot plate test was performed to determine the central analgesic activity of MECC. The test was carried out using Eddy’s hot plate (Kshitij Innovations, Haryana, India). Hot plate temperature was kept constant at 50 ± 0.5 °C. The mice were gently placed on the hot plate and jumping, withdrawal of paw(s), forepaw licking were considered as nociception. The mice were kept on the hot surface only for the 20 s (cut-off time) to avoid any thermal injury [37]. A latency was determined before treatment as a baseline for the study. After vehicle, standard drug morphine (i.p.) or MECC (p.o.) treatment the latency periods were measured at 30, 45, 60, 90 and 120 min. The maximal possible effect (MPE) in % of individual mice was measured by following formula: % MPE = [(post-treatment latency – pre-treatment latency)/(cut-off time – pre-treatment latency)] × 100.

#### Tail immersion test

The test was carried out based on the findings that morphine-like drugs extend the tail withdrawal time from hot water in mice [38]. Mice were gently restrained using ‘Chux’ and one to two cm of the tail was immersed into hot water set at 52 ± 1 °C. The latency between submersion and withdrawal of the tail was recorded and considered as the index of nociception. Mice that demonstrated withdrawal of tail between 1.5 and 3.5 s from the hot water were selected for the test. A 20s cut-off period was maintained to avoid tissue damage of tail of mice. The latency time was recorded before and after 30, 45, 60, 90 and 120 min of the vehicle, morphine (i.p.) or MECC (p.o.) treatment. The % MPE was calculated from pre and post treatment latency as described in hot plate test.

#### Acetic acid-induced writhing test

The acetic acid-induced writhing test was conducted to determine the central and peripheral antinociceptive effect of MECC against chemical induced nociception. Mice were treated (i.p.) with 0.6 % (v/v) acetic acid to induce writhing. Acetic acid was employed at the dose of 10 mL/kg (b.w.) 30 min after vehicle or MECC and 15 min of diclofenac sodium (i.p.) administration. Mice were then placed in separate box. After 5 min of acetic acid treatment, the number of writhing was recorded for 30 min [39]. The writhing was defined by contraction of the abdomen, arching of back, twisting of the trunk and/or pelvis ending with the elongation of limbs.

#### Formalin-induced licking test

A volume of 20 μl of formalin (2.5 % formalin (0.92 % formaldehyde) prepared in saline) was injected into the subplantar region of the right hind paw of mice to induce pain. Formalin was injected 15 min after morphine and 30 min after vehicle or MECC treatment. Responses like licking, biting, lifting, shaking of right hind paw were considered as nociception. The nociceptive responses were measured 0 to 5 min (early phase) and 15 to 30 min (late phase) following formalin treatment, representing to the neurogenic and inflammatory pain responses respectively [40].

#### Glutamate-induced paw licking and edema test

Mice were injected with 20 μl of 20 μ mol glutamate (prepared in saline, pH 7.4) in the sub-plantar area of the right hind paw. Mice received vehicle or MECC and diclofenac sodium 30 and 15 min before glutamate injection respectively. The right hind paw thickness of mice was measured before glutamate challenge using digital slide caliper (M: 091552; Shanghai Shenhan Measuring Tools Co., Ltd, Shanghai, China). The licking was measured for 15 min after glutamate challenge and accounted as indicative of nociception. Then paw thickness of right hind paw of every mouse was measured again and the degree of edema (Δ) in mm was calculated from the following formula: Δ = (paw thickness after treatment – paw thickness before treatment) [41].

#### Analysis of the possible mechanism of action of MECC

##### Involvement of the opioid system

In order to determine the participation of opioid system in the pain inhibition effect of MECC naloxone was intraperitoneally administered 15 min before MECC (50, 100, 200 and 400 mg/kg) or morphine sulfate administration in the hot plate and tail immersion test [42]. The latencies of the hot plate and tail immersion were sequentially recorded at pre-treatment, 30, 45, 60, 90, 120 min of after administration of MECC or morphine and assembled with the result of the hot plate and tail immersion test respectively. The same cut-off period of the 20 s was maintained for any thermal injury.

#### Involvement of the cyclic guanosine monophosphate (cGMP) pathway

Mice were pre-treated with methylene blue (MB), a non-specific guanylylcyclase/NO inhibitor to verify the possible involvement of cGMP in the pain inhibition action of MECC. MB (20 mg/kg) was administered (i.p.) 15 min before the employment of effective doses (100, 200, 400 mg/kg) of MECC. After 30 min of treatment, animals were intraperitoneally administered 0.6 % (v/v) acetic acid at the dose of 10 mL/kg to induce writhing. The nociceptive behavior was recorded for 30 min, starting after 5 min of acetic acid treatment. The abdominal writhing syndrome was counted as an indication of nociceptive behavior [43, 44].

#### Involvement of the ATP-sensitive K^+^ channel pathway

The participation of K^+^ channel in the antinociceptive action of the effective doses of MECC (100, 200 and 400 mg/kg was evaluated to the groups of mice, pre-treated with glibenclamide, an ATP-sensitive K^+^ channel inhibitor. Glibenclamide was intraperitoneally administered at the dose of 10 mg/kg 15 min before the treatment of MECC. After 30 min of above treatments, the mice of each group were challenged with 0.6 % acetic acid (i.p.) to induce writhing. After 5 min of an acetic acid challenge, the number of writhing was noted for 30 min as an indication of nociceptive response [44, 45].

### Statistical analysis

Data are presented as mean ± standard error of mean (SEM). Statistical analysis of the results was performed using one-way analysis of variance (ANOVA). The significance of differences between groups was tested by Dunnett’s or Bonferroni’s test, as appropriate and *p* < 0.05 values were considered as significant. SPSS 22 software was used for performing the statistical analysis. IC_50_ values were calculated using GraphPad prism (version 6.01) software.

## Results

### Phytochemical analysis

**Fig. 1:**
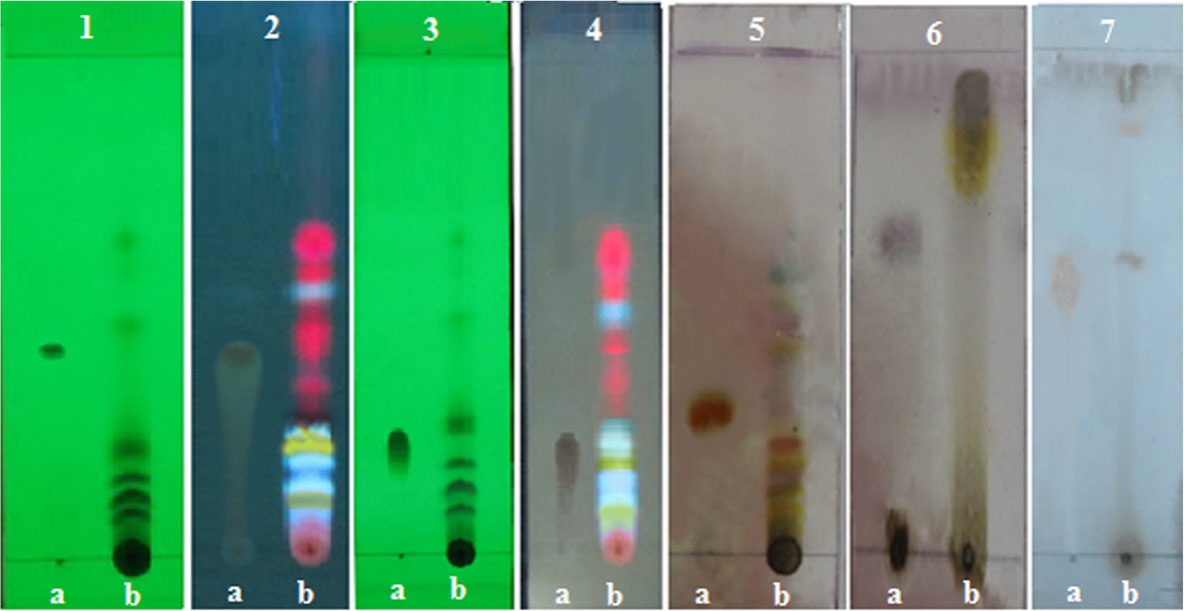
Thin layer chromatograms of methanol extract of *C. cristata* (MECC). Experiment 1- (at 254 nm, a: quercetin, b: MECC), 2-(at 356 nm, a: quercetin, b: MECC) for flavonoids (orange, yellow spots); 3- (at 254 nm, a: gallic acid, b: MECC), 4- (at 356 nm, a: gallic acid, b: MECC) for phenolics (fluorescence blue spots); 5- after spray of 1 % vanillin-H_2_SO_4_ reagent (a: stigmasterol, b: MECC) for steroids (purple spots); 6- after spray of H_2_SO_4_ (a: digoxin, b: MECC) for glycosides (dark-violet spots); 7- after spray of Dragendroff’s reagent (a: atropine, b: MECC) for alkaloids (light pink spots)

**Fig. 2:**
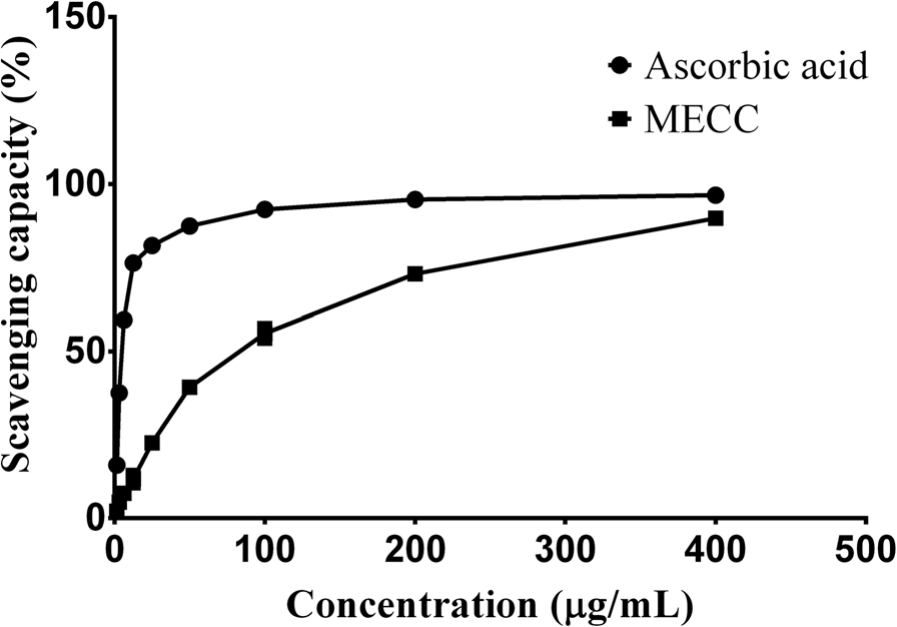
DPPH free radical scavenging capacity (%) of ascorbic acid and methanol extract of *C. cristata* at various concentrations (μg/mL). Each data point represents mean ± SEM (*n* = 3)

Phytochemical analysis of the crude methanol extract of *C. cristata* confirmed the presence of carbohydrates, alkaloids, flavonoids, tannins, steroids, proteins, saponins, glycosides and resins. The major phytochemical composition of MECC was further confirmed by TLC analysis. TLC fingerprinting of MECC (Fig. 1) indicate the presence of flavonoids (yellow or orange spots), phenolics (blue fluorescence spots), steroids (purple spots), glycosides (dark-violet after applying spray reagents) and alkaloids (pink spots). The comparison of R_f_ values between standard marker and extract spots are presented in Table 1. From the quantitative analysis of MECC, total phenolic and flavonoid content were estimated as 45.21 ± 1.86 mg_GAE_/g_extract_ and 66.69 ± 2.14 mg_QE_/g_extract_ respectively. In DPPH assay the extract showed dose-dependent free radical scavenging activity (Fig. 2). The antioxidant activity of ascorbic acid and MECC in DPPH assay was estimated with the IC_50_ value of 5.14 ± 0.08 μg/mL and 76.34 ± 2.36 μg/mL respectively.

**Table 1:** Phytochemical analysis of MECC by TLC

Phytochemicals	Color	Retention factor (R_f_)	
		Extract	Standard
Flavonoids	Yellow, orange^a^	1.02, 0.20	0.41
Phenolics	blue fluroscence^a^	0.04, 0.10, 0.14, 0.18, 0.47	0.22
Steroids	Purple^b^	0.12, 0.13, 0.16	0.34
Glycosides	Dark-violet^b^	0.94	0.60
Alkaloids	Light pink^b^	0.58, 0.83	0.54

R_f_ values are the mean of two chromatograms

^a^ Represents the color was observed under the visible or UV light

^b^ Represents the color was observed after applying the appropriate spray reagent. The standard for flavonoids, phenolics, steroids, glycosides and alkaloids was quercetin, gallic acid, stigmasterol, digoxin, and atropine respectively

### Acute toxicity

Oral administration of MECC at 1000–4000 mg/kg doses did not cause any mortality as well as allergic reactions, salivation, convulsion, tremors, diarrhea or behavioral changes during the observation period. In addition, no statistically significant gross changes or abnormality of the vital organs of the mice were observed between control and experimental groups (Table 2).

**Table 2:** Effect of oral administration of MECC on gross changes to the vital organs of mice

Treatment	Organ weight (g) (Organ body index in %)					
	Body weight	Heart	Kidneys	Liver	Lungs	Spleen
Vehicle	22.04 ± 0.25	0.15 ± 0.01 (0.66)	0.38 ± 0.01 (1.72)	1.32 ± 0.02 (6.01)	0.23 ± 0.02 (1.03)	0.10 ± 0.01 (0.46)
MECC (1000 mg/kg)	21.28 ± 0.16	0..11 ± 0.01 (0.52)	0.32 ± 0.02 (1.48)	1.33 ± 0.13 (6.23)	0.18 ± 0.01 (0.86)	0.07 ± 0.01 (0.35)
MECC (2000 mg/kg)	23.39 ± 0.48	0.18 ± 0.01 (0.79)	0.44 ± 0.03 (1.85)	1.79 ± 0.12 (7.78)	0.27 ± 0.02 (1.14)	0.15 ± 0.02 (0.63)
MECC (4000 mg/kg)	23.27 ± 0.58	0.17 ± 0.01 (0.89)	0.41 ± 0.03 (1.74)	1.76 ± 0.19 (7.52)	0.29 ± 0.04 (1.23)	0.14 ± 0.02 (0.60)

Each value is presented as the mean ± SEM (*n* = 5). MECC = Methanol extract of *C. cristata*, organ body index (%) = (weight of organ/weight of body)/100. *P* is not ≤ 0.05, compared to control group (Dunnett’s test)

### Hot plate test

**Fig. 3:**
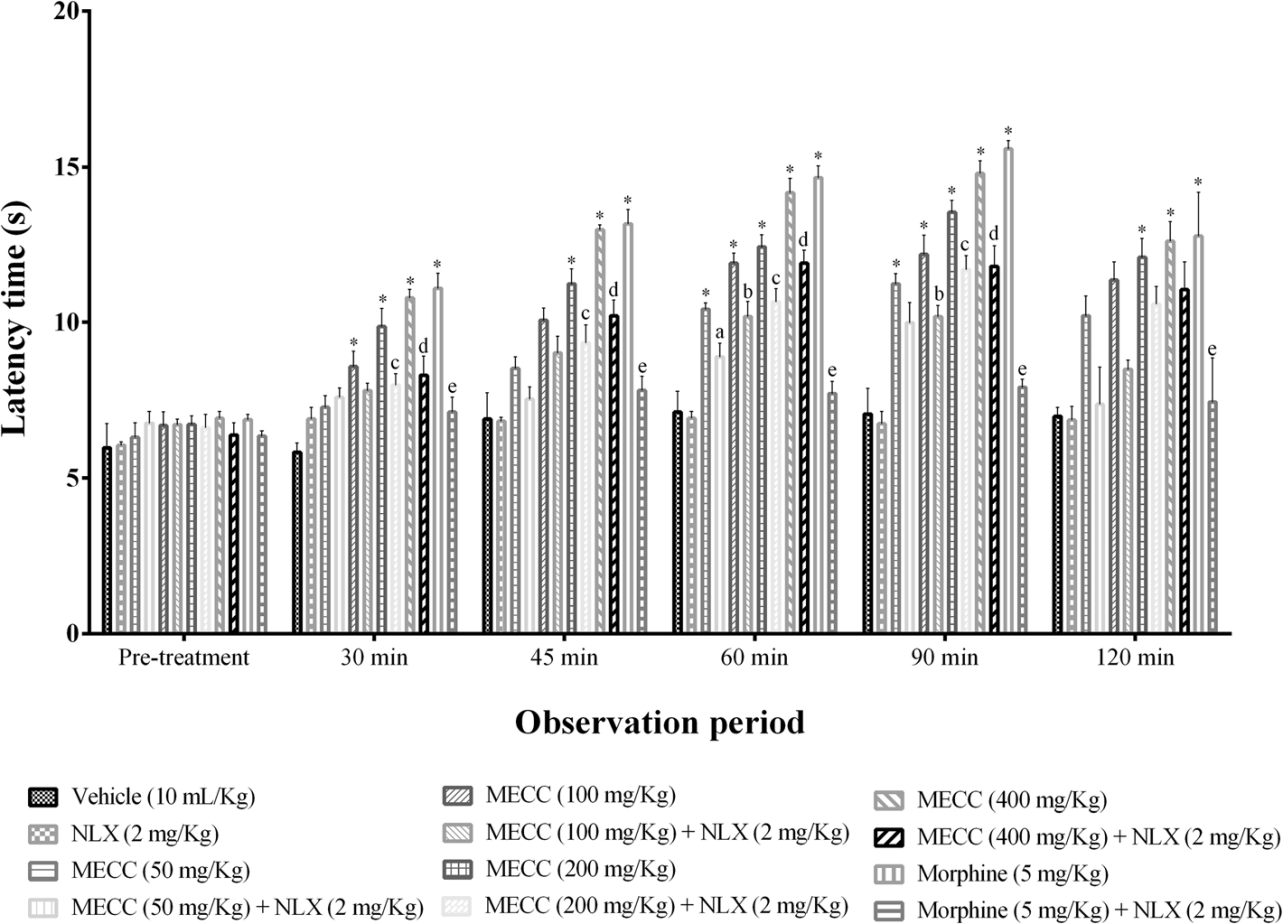
Effect of methanol extract of *C. cristata* in hot plate test. Data are presented as the mean ± SEM (*n* = 5). MECC = Methanol extract of *C. cristata*; NLX = Naloxone. * represents *p* < 0.001 compared with the control group (Dunnett’s test). ^a, b, c, d, e^ represents *p* < 0.001, compared with the MECC 50, MECC 100, MECC 200, MECC 400, morphine, group respectively (Bonferroni’s test)

Oral treatment of MECC in mice caused a significant (*p* < 0.001) increase of latency period in hot plate test at all experimental doses compared to control group (Fig. 3). The effect was maximum (14.79 ± 0.41 s) at 400 mg/kg and dose related. The highest reaction time against thermal stimuli for 50, 100 and 200 mg/kg of MECC was 11.24 ± 0.23, 12.19 ± 0.61, 13.54 ± 0.39 s respectively. MECC produced significant (*p* < 0.001) % MPE at the doses of 100, 200 and 400 mg/kg throughout the observational period. The highest inhibition of thermal pain of MECC (60.06 % at the dose of 400 mg/kg) and morphine (66.27 %) was observed at 90 min. However, standard drug, morphine produced maximum latent time as well as %MPE at all the observation period (Table 3). The mean latent period for naloxone was 6.72 ± 0.15 s. Naloxone could not produce any significant effect itself compared to control group (6.65 ± 0.26 s). On the other hand, naloxone significantly (*p* < 0.001) reversed the activity of morphine and MECC at the all experimental doses.

**Table 3:** The percentage of maximum possible effect (%MPE) of MECC in hot plate test

Treatment	Dose (mg/kg)	%MPE				
		30 min	45 min	60 min	90 min	120 min
Vehicle	-	-2.49 ± 6.47	5 ± 9.24	7.44 ± 5.44	5.17 ± 11.70	5.73 ± 6.69
NLX	2 (i.p.)	6.06 ± 2.50	5.51 ± 1.26	6.11 ± 2.00	4.98 ± 2.68	5.73 ± 3.64
MECC	50	6.97 ± 1.42	15.66 ± 4.85	29.61 ± 3.22*	36.02 ± 1.36*	28.39 ± 4.16
MECC	100	14.19 ± 3.03	25.32 ± 2.50*	39.07 ± 1.87*	40.98 ± 5.99*	35.26 ± 3.02*
MECC	200	23.64 ± 3.90*	34.04 ± 3.14*	43.02 ± 2.17*	51.43 ± 2.24*	40.01 ± 5.62*
MECC	400	29.62 ± 1.23*	46.24 ± 1.89*	55.36 ± 3.67*	60.06 ± 3.28*	43.48 ± 4.83*
Morphine	5 (i.p.)	32.07 ± 3.64*	47.89 ± 3.68*	59.14 ± 3.26*	66.27 ± 2.18*	45.24 ± 10.25*

Each value is presented as the mean ± SEM (*n* = 5). MECC = Methanol extract of *C. cristata*; NLX = Naloxone. * represents *p* < 0.001 compared with the control group (Dunnett’s test)

### Tail immersion test

**Fig. 4:**
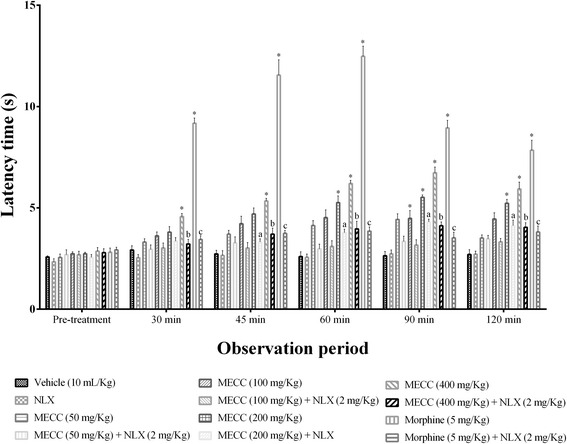
Effect of methanol extract of *C. cristata* in tail immersion test. Data are presented as the mean ± SEM (*n* = 5). MECC = Methanol extract of *C. cristata*; NLX = Naloxone. * represents *p* < 0.001 compared with the control group (Dunnett’s test). ^a, b, c^ represents *p* < 0.01, compared with the MECC 200, MECC 400, morphine group respectively (Bonferroni’s test)

MECC significantly increased hot water latency time in tail immersion test at the 100, 200 and 400 mg/kg test doses (*p* < 0.001) in a dose-dependent way (Fig. 4). MECC showed the highest latency on hot-water at the dose of 400 mg/kg throughout the experimental periods and the effect was maximum on the 90 min (6.73 ± 0.29 s). However, the significant maximal latency for the dose of 100 and 200 mg/kg of MECC was also found on 90 min as 4.48 ± 0.39 s and 5.52 ± 0.12 s respectively. MECC showed maximum 22.60 % inhibition on the 90 min where morphine showed 56.30 % inhibition (latency 12.48 ± 0.50 s) on the 60 min of the experimental period. The experimental doses of MECC produced significant (*p* < 0.001) %MPE at different observation period (Table 4). Morphine produced maximum latency as well as %MPE at all the experimental periods (*p* < 0.001). The reversal effect of naloxone was also observed. Naloxone significantly (*p* < 0.01) antagonized the pain inhibition activity of morphine and MECC at the doses of 200 and 400 mg/kg.

**Table 4:** The percentage of maximum possible effect (%MPE) of MECC in tail immersion test

Treatment	Dose (mg/kg)	%MPE				
		30 min	45 min	60 min	90 min	120 min
Vehicle	-	1.95 ± 1.15	0.87 ± 0.83	0.10 ± 1.44	0.32 ± 1.07	0.71 ± 1.20
NLX	2 (i.p.)	1.12 ± 1.18	1.74 ± 1.99	1.25 ± 1.33	2.43 ± 1.42	2.09 ± 0.74
MECC	50	4.35 ± 0.57	6.55 ± 0.97	8.99 ± 1.60	10.77 ± 1.38*	5.40 ± 1.56
MECC	100	5.04 ± 1.18	8.57 ± 2.17	10.28 ± 2.11	10.05 ± 2.31*	9.88 ± 1.88
MECC	200	6.15 ± 1.56	11.36 ± 1.65	14.65 ± 1.71*	16.12 ± 0.58*	14.39 ± 1.32*
MECC	400	9.97 ± 1.02*	14.46 ± 0.95*	19.51 ± 0.56*	22.60 ± 1.33*	17.91 ± 1.79*
Morphine	5 (i.p.)	36.98 ± 1.39*	50.92 ± 4.19*	56.30 ± 2.72*	35.65 ± 2.07*	29.17 ± 3.41*

Each value is presented as the mean ± SEM (*n* = 5). MECC = Methanol extract of *C. cristata*; NLX = Naloxone. * represents *p* < 0.001, compared with the control group (Dunnett’s test)

### Acetic acid-induced writhing test

MECC at the doses of 100, 200 and 400 mg/kg and diclofenac sodium significantly (*p* < 0.001) reduced the number of writhing induced by acetic acid compared to control group in mice (Table 5). Oral administration of MECC caused the highest reduction of the number of writhing (26.50 ± 1.55) at the doses of 400 mg/kg. The highest inhibition of writhing of MECC was 55.76 % compared to the control group and the percentage of inhibition of writhing was dose dependent. Standard drug (diclofenac sodium) caused a minimum number of writhing (18.80 ± 1.02) and showed the maximum inhibition of 68.61 %.

**Table 5:** Effect of methanol extract of *C. cristata* in the acetic acid-induced writhing test

Treatment	Dose (mg/kg)	Number of Writhing	% Inhibition
Vehicle	-	59.90 ± 1.03	-
Diclofenac sodium	10 (i.p.)	18.80 ± 1.02*	68.61
MECC	50	51.70 ± 1.62	13.69
MECC	100	36.60 ± 1.77*	38.89
MECC	200	34.90 ± 1.13*	41.74
MECC	400	26.50 ± 1.55*	55.76

Each value is presented as mean ± SEM (*n* = 5), MECC = Methanol extract of *C. cristata*. * represents *p* < 0.001 compared with the control group (Dunnett’s Test)

### Formalin-induced licking

**Fig. 5:**
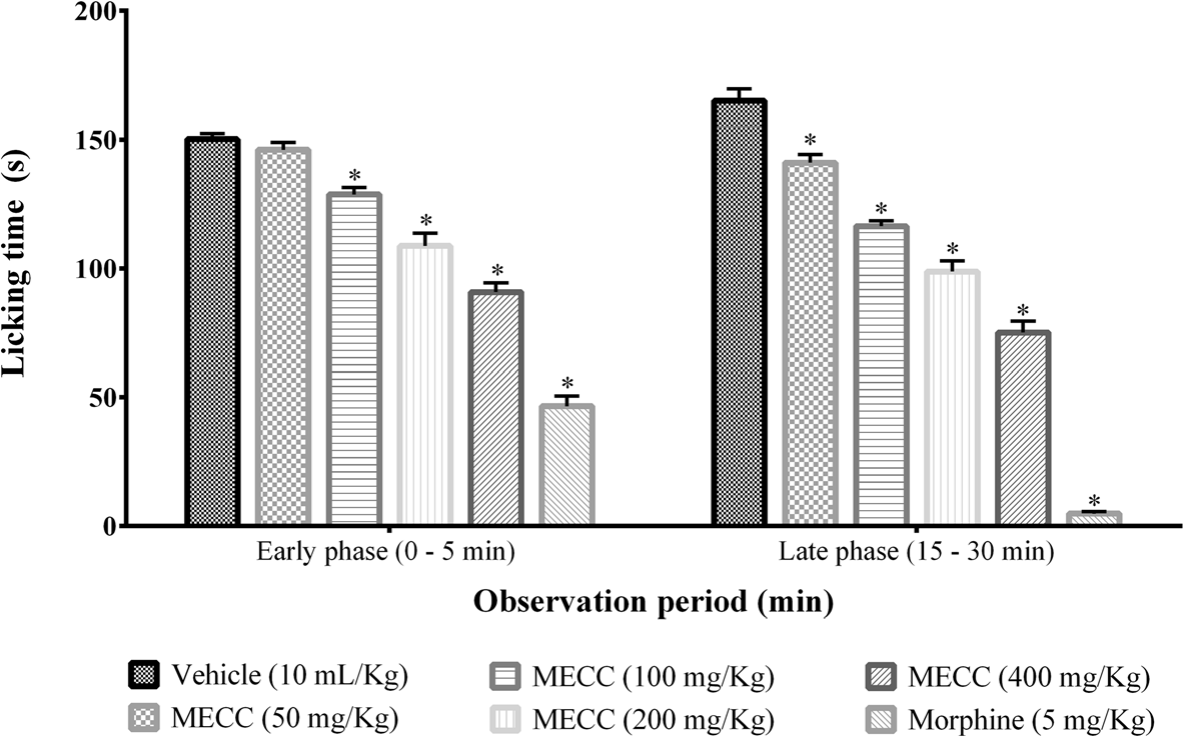
Effect of methanol extract of *C. cristata* in formalin-induced paw licking test. Data are presented as mean ± SEM (*n* = 5), MECC = Methanol extract of *C. cristata*. * represents *p* < 0.001 compared with the control group (Dunnett’s Test)

MECC produced marked inhibition (*p* < 0.001) of formalin-induced licking at the doses of 100, 200, 400 mg/kg in the early phase and at all the test doses in the late phase compared to control group (Fig. 5). The lowest spent time in licking both in early (90.78 ± 3.61 s) and late phase (75.10 ± 4.46 s) was observed at the dose of 400 mg/kg compared to other doses of MECC where morphine caused minimum licking in mice from early phase (46.53 ± 4.05 s) to late phase (4.82 ± 0.84 s). The inhibition response of MECC was dose dependent in both phases. Besides, the late phase licking inhibition of the test by morphine and MECC was more prominent. MECC and morphine exerted the highest inhibition of licking by 54.50 and 97.08 % respectively in the late phase.

### Glutamate-induced paw licking and edema

**Fig. 6:**
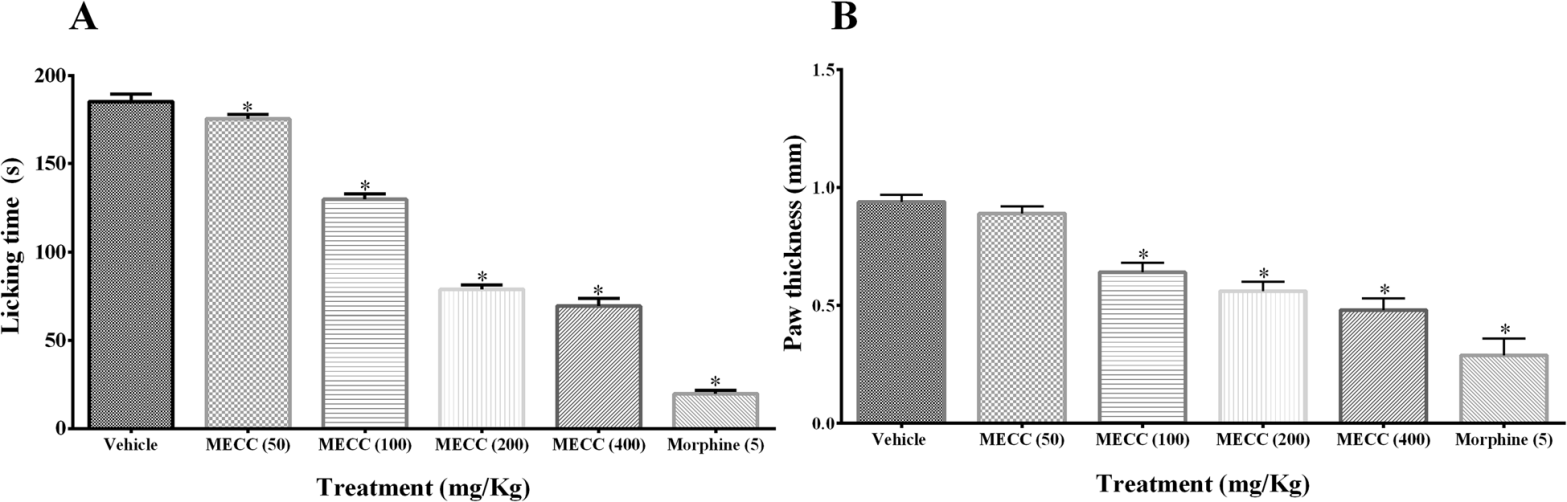
Effect of methanol extract of *C. cristata* in glutamate-induced paw licking (**a**) and edema test (**b**). Data are presented as mean ± SEM (*n* = 5), MECC = Methanol extract of *C. cristata*. * represents *p* < 0.001 compared with the control group (Dunnett’s Test)

The treatment of diclofenac sodium and 100, 200 and 400 mg/kg doses of MECC significantly (*p* < 0.001) decreased the glutamate-induced paw licking as well as paw edema in mice (Fig. 6). The reduction of paw licking time and edema by MECC in mice was dose reliant as stronger at 400 mg/kg (69.32 ± 4.22 s and 0.48 ± 0.05 mm respectively) compared to control group (185.33 ± 4.20 s and 0.94 ± 0.03 mm respectively). MECC produced the highest reduction of glutamate-induced paw licking and edema at the dose of 400 mg/kg by 62.59 and 49.14 % respectively. However, diclofenac sodium showed the maximum inhibition of paw licking and edema by 74.05 and 65.81 % respectively.

### Involvement of the cyclic guanosine monophosphate (cGMP) pathway

**Fig. 7:**
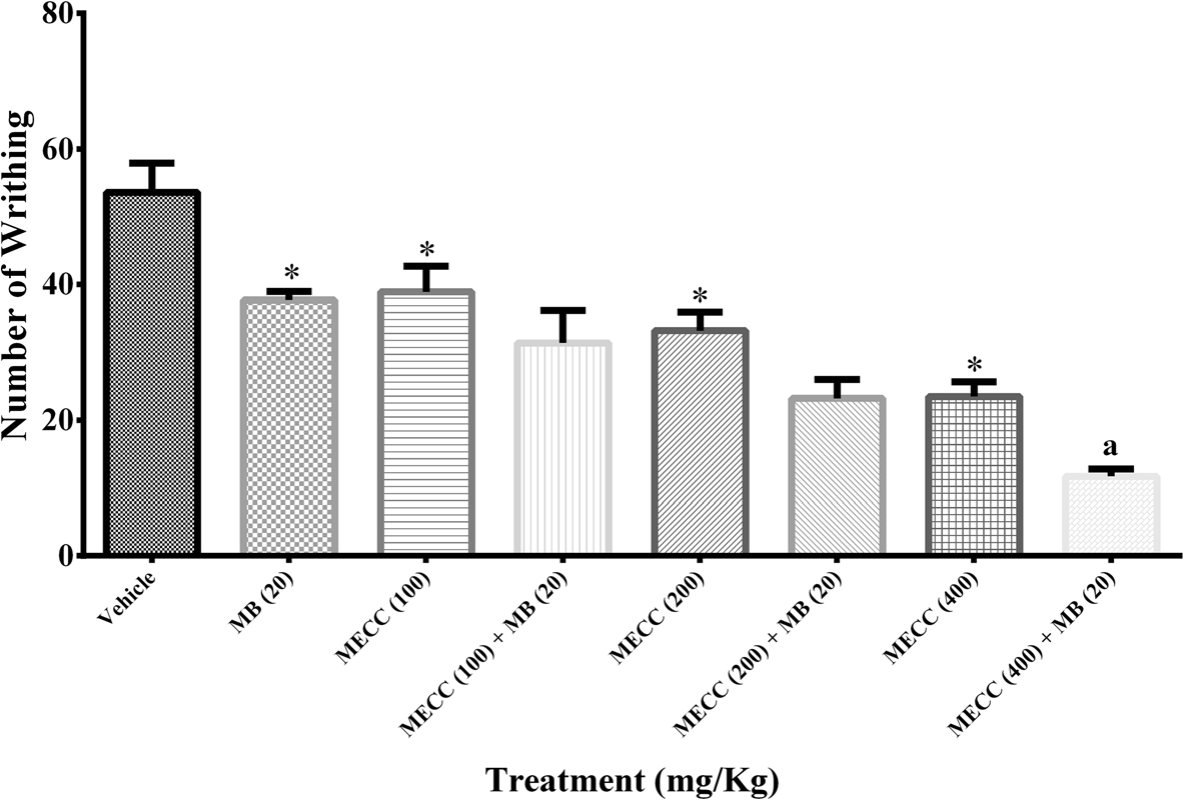
Effect of methanol extract of *C. cristata* on the involvement of the cyclic guanosine monophosphate (cGMP) pathway. Data are presented as mean ± SEM (*n* = 5), MECC = Methanol extract of *C. cristata*. MB = methylene blue. * represents *p* < 0.001 compared with the control group (Dunnett’s Test). ^a^ represents *p* < 0.05 compared with the MECC 400 group (Bonferroni’s test)

As presented in Fig. 7, MECC (100, 200 and 400 mg/kg) and methylene blue (20 mg/kg) in alone caused significant inhibition of number of writhing (38.90 ± 3.77, 33.20 ± 2.75, 23.50 ± 2.17 and 37.70 ± 1.23 respectively) compared to control group (53.60 ± 4.31). The MB treatment with 100, 200 and 400 mg/kg doses of MECC amplified the antinociceptive activity by reduction of the number of writhing (31.40 ± 4.80, 23.20 ± 2.84, 11.70 ± 1.06 respectively). The methylene blue and MECC (400 mg/kg) together significantly (*p* < 0.05) enhanced the antinociceptive effect compared to the treatment with methylene blue and MECC (400 mg/kg) alone.

### Involvement of the ATP-sensitive K^+^ channel pathway

**Fig. 8:**
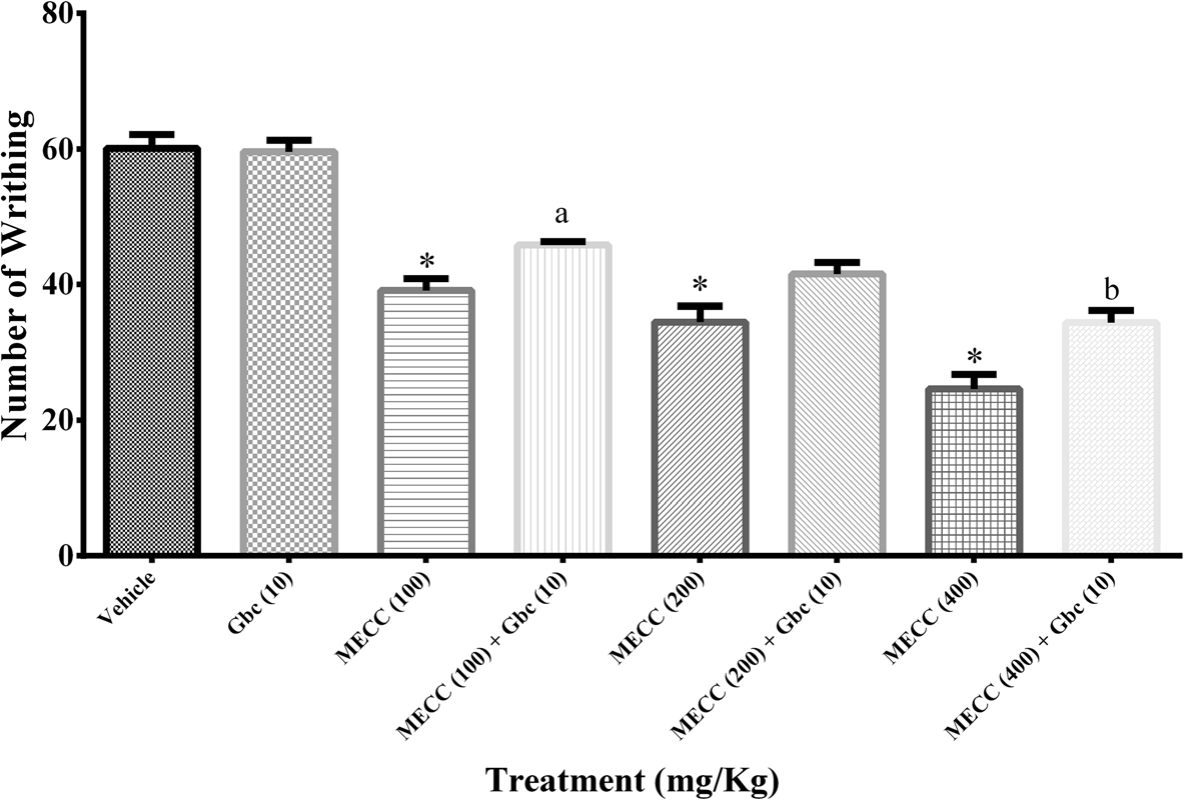
Effect of methanol extract of *C. cristata* on the involvement of the ATP-sensitive K^+^ channel pathway. Data are presented as mean ± SEM (*n* = 5), MECC = Methanol extract of *C. cristata*. Gbc = glibenclamide. * represents *p* < 0.001 compared with the control group (Dunnett’s Test). ^a, b^ represents *p* < 0.05 compared with the MECC 100, MECC 400 group respectively (Bonferroni’s test)

The action of glibenclamide and MECC on the acetic acid-induced writhing response in mice are presented in Fig. 8. Glibenclamide did not cause any significant alteration of the number of writhing response (59.60 ± 1.69) compared to control group (60.10 ± 2.03). MECC produced significant inhibition of writhing response at all the tested doses (*p* < 0.001). However, glibenclamide reversed the antinociceptive action when administered with MECC (100, 200, 400 mg/kg). The reversal effect was significant (*p* < 0.05) for the glibenclamide plus MECC 100 (45.80 ± 0.51) as well as glibenclamide plus 400 mg/kg (34.40 ± 1.80) treatment, compared to the treatments with MECC 100 (39.10 ± 1.76) and 400 mg/kg (24.60 ± 2.16) alone respectively.

## Discussion

The current investigation demonstrates that oral treatment of MECC inhibited thermal and chemical-induced pain threshold and produced dose-dependent central and peripheral antinociceptive effect in different pain models. In acute toxicity study, no mortality, allergic reactions, salivation, convulsion, tremors, diarrhea, behavioral and gross changes of vital organs in mice after the administration of 10-fold higher dose than the maximum experimental dose of MECC suggest that MECC was non-toxic at our experimental doses.

The significant (*p* < 0.001) increase of latency time and inhibition of thermal pain threshold of mice in the hot plate (Fig. 3) and tail immersion (Fig. 4) tests at different doses indicate that MECC has a profound central antinociceptive effect. These tests are widely used to evaluate the central antinociceptive effect of drugs and the tail withdrawal response determines the antinociceptive effect of centrally acting analgesics only [46]. Therefore, the antinociceptive activity of MECC in tail immersion test further approve the outcome of hot plate test. Besides, hot plate and tail immersion evaluate μ_1_-_,_ δ_1_- κ_3_- and σ_2_ - opioid receptors mediated supraspinal reflex and μ_2_-, κ_1_- and δ_2_- opioid receptors mediated spinal reflexes respectively [47–50]. Our findings indicate that central antinociceptive effect of MECC is due to its action on supraspinal as well as the spinal system. Naloxone significantly reversed the antinociceptive effect of MECC at different doses level in both heat-induced pain models. This action further confirms the involvement of opioid receptors mediated central antinociceptive activity of MECC.

The writhing nociceptive response was significantly (*p* < 0.001) inhibited by MECC in acetic acid-induced writhing test (Table 5). The test is widely used for the determination of central and peripheral antinociceptive activity of new substances [51]. Intraperitoneal treatment of acetic acid increases the degree of endogenous prostaglandins (PGs), serotonin, histamine, bradykinin, substance P, cyclooxygenase (COX), lipooxygenase (LOX) and cytokines such as IL-8, IL-1β, and TNF-α in the peripheral fluid tissue. These endogenous inflammatory mediators and cytokines then enter into the dorsal horn of central nervous system and stimulate the primary afferent nociceptors [52]. This action results in the induction of inflammatory pain and writhing syndrome [53]. Therefore, significant inhibition of acetic acid-induced writhing by MECC can be attributed to the suppression of release of peripheral inflammatory mediators as well as interruption of the signal transduction of primary afferent nociceptors.

The pain induced by formalin in mice paw is mediated by two distinct pathways. First, the early phase (immediately after formalin injection), characterized by neurogenic pain, is induced due to direct stimulation of sensory afferent fibers as well as activation of C-fibers. Bradykinin and substance P are also involved in the inducement of nociception in this phase. Second, the late phase (15 min after formalin injection), where the inflammatory pain is produced by the action of prostaglandins (PGs), bradykinin, serotonin and histamine-like inflammatory mediators in peripheral tissues [54, 55]. In addition, formalin induced late phase pain is also caused by the functional changes in the dorsal horn of the spinal cord [56]. The result of the formalin-induced paw licking test (Fig. 5) shows that morphine and MECC significantly (*p* < 0.001) inhibited the nociception of both phases. The inhibition was dose dependent and more prominent in the late phase. Central analgesics (opioids) inhibit both phases whereas peripheral analgesics (aspirin, hydrocortisone) inhibit mainly the late phase of formalin-induced paw-licking in mice [57–59]. Therefore, significant inhibition of both phase paw lickings in formalin test further indicates the central pain prevention activity of MECC and supports the outcome of the hot plate and tail immersion tests. Besides, the late phase paw-licking deterrence suggests the inhibition of the inflammatory mediators, which was observed in the acetic acid-induced writhing test.

The glutamate-induced paw licking nociception is mediated via NMDA (N-methyl-D-aspartate) receptors while edema formation is accompanied by non-NMDA receptors (AMPA, Kainate) in peripheral, supraspinal and spinal sites. Glutamate release substance P and IL-1β, TNF- α like pro-inflammatory cytokines for the transmission of pain signals from peripheral nervous system to the dorsal horn of the central nervous system. Furthermore, the pro-inflammatory signals involve the stimulation of TNF- α, IL-1, IL-6 genes by nitric oxide synthase (NOS) and reactive oxygen species (ROS) [60–62]. MECC significantly (*p* < 0.001) reduced glutamate-induced paw licking and edema (Fig. 6). The result suggests that MECC is involved in the suppression of NMDA, non-NMDA receptors as well as disruption of ROS, NOS mediated pro-inflammatory signals. Besides, *C. cristata* and its isolated compound, HA, have been reported to inhibit the ROS and NOS, IL-1 induced pro-inflammatory cytokine TNF-α respectively [63–66] which strongly suggests the association of NOS, ROS as well as TNF-α mediated pro-inflammatory signal interruption activity of MECC.

The present study examined the association of cGMP pathway in pain inhibition activity of MECC. It has been reported that NO associated nociceptive signal transduction is influenced by the cellular level of cGMP, where the concentration of cGMP in the intracellular level is controlled by the action of sGC (soluble guanylyl cyclase) [43]. The involvement of cGMP in MECC induced antinociception was evaluated by treating the mice with methylene blue (MB), a guanylyl cyclase inhibitor, before the inducement of acetic acid treated abdominal pain. The results indicated that (Fig. 7) methylene blue significantly enhanced the pain inhibition activity of MECC as well as inhibited the nociceptive response alone. MB interrupt the NO associated nociceptive signal transduction by the inhibition of the action of sGC as well as the availability of cGMP in intracellular level and promotes antinociceptive effect [43]. As MB pre-treatment enhanced the antinociceptive activity of MECC compared to the MECC alone, it can be suggested that pain inhibition action of MECC involves cGMP pathway. The outcomes of the present investigation also suggest the involvement of the ATP-sensitive K^+^ channel pathway in the antinociception of MECC. Glibenclamide, an ATP-sensitive K^+^ channel blocker, partly abolished the pain inhibition activity shown by MECC (Fig. 8). It has been reported that glibenclamide cause the blockade of ATP-sensitive K^+^ channel without affecting the voltage-gated and Ca^2+^ activated K^+^ channels [67, 68]. Therefore, the result indicates that antinociceptive action of MECC could be related to the subsequent efflux of K^+^ ions by the opening of the ATP-sensitive K^+^ channel as well as reduction of the membrane excitability by hyperpolarization and/or repolarization [69].

Phytochemical study of *C. cristata* reveals the presence of alkaloids, flavonoids, tannins, steroids, glycosides. TLC analysis of MECC further confirms the presence of these phyto-compounds. The amount of total phenolic, flavonoid and antioxidant activity against DPPH free radicals of MECC was considerable. The NMDA signaling in the pain inducement is influenced by the free radicals [69]. Therefore, pain inhibition action of MECC could be partially credited to the action of MECC against free radicals. Plant containing flavonoids have been reported to inhibit the pro-inflammatory cytokines like TNF-α, IL-6 [70]. Besides, the plant having alkaloids, flavonoids and tannins possess significant analgesic property [71–73]. Therefore, it can be suggested that phytochemicals, particularly phenolics, flavonoids, tannins and alkaloids present in MECC may contribute to its antinociceptive activity.

## Conclusion

Considering the results of different pain models in mice, it can be suggested that crude methanol extract of *C. cristata* possess strong and dose-dependent antinociceptive activity. The effect is associated with opioid receptor, cGMP pathway, ATP-sensitive K^+^ channel as well as suppression of peripheral mediators such as PGs, COX, LOX, NMDA, non-NMDA receptors. The outcomes justify the ethnomedicinal implication of *C. cristata* in different painful conditions such as headache, eye inflammations, sores, ulcers, skin eruption, wounds and body swelling. However, further studies regarding direct modulation of the nociceptive mediators and isolation of active principle(s) responsible for observed pharmacological actions are required to elucidate precise mechanisms of this plant. The findings of the present study suggest that *C. cristata* possibly contain potential bioactive molecules which could be used for the development of the analgesic lead compound.

## Abbreviations

AMPA: Alpha-amino-3-hydroxy-5-methylisoxazole-4-propionic acid
ANOVA: One-way analysis of variance
ATP: Adenosine triphosphate
AVMA: American Veterinary Medical Association
b.w.: Body weight
BPH: Benign prostate hyperplasia
cGMP: Cyclic guanosine monophosphate
COX: Cyclooxygenase
DPPH: 2,2-Diphenyl-1-picrylhydrazyl
GAE: Gallic acid equivalents
Gbc: Glibenclamide
HA: Hyaluronic acid
i.p.: Intraperitoneal
IC_50_: Half maximal inhibitory concentration
icddr,b: International Center for Diarrhoeal Disease Research, Bangladesh
IL: Interleukin
IL-1 β: Interleukin-1 beta
LOX: Lipooxygenase
MB: Methylene blue
MECC: Methanol extract of *Celosia cristata*
MPE: Maximal possible effect
NLX: Naloxone
NMDA: N-methyl-D-aspartate
NOS: Nitric oxide synthase
OECD: Organization for Economic Cooperation and Development
p.o.: Per oral
PGs: Prostaglandins
QE: Quercetin equivalents
r.p.m.: Rotation per minute
R_f_: Retention factor
ROS: Reactive oxygen species
SEM: Standard error of mean
sGC: Soluble guanylyl cyclase
TLC: Thin layer chromatography
TNF-α: Tumor necrosis factor-alpha
TPC: Total phenolic content
